# The Effect of Weekend Surgery on Outcomes of Emergency Laparotomy: Experience at a High Volume District General Hospital

**DOI:** 10.7759/cureus.23537

**Published:** 2022-03-27

**Authors:** Maitreyi S Patel, Joel J Thomas, Xavier Aguayo, Daniel Gutmann, Sayed Haschmat Sarwary, Mehmood Wain

**Affiliations:** 1 Surgery, Barking, Havering and Redbridge University Hospitals NHS Trust, Romford, GBR; 2 General Surgery, Barking, Havering, and Redbridge University Hospitals NHS Trust, Romford, GBR; 3 Surgery, Barking, Havering, and Redbridge University Hospitals NHS Trust, Romford, GBR

**Keywords:** mortality, morbidity, emergency surgery, outcomes, weekend, emergency laparotomy

## Abstract

Aims

Emergency laparotomies (ELs) are associated with significant morbidity and mortality. Delays to the theater are inevitably associated with worse outcomes. Higher mortality has been reported with admissions over the weekend. The aim of this study is to compare the delays and outcomes of emergency laparotomies performed on weekdays (WD) and weekends (WE) at a high-volume, large district general hospital.

Methods

A retrospective review of a prospectively maintained database was performed for all patients who underwent general surgical emergency laparotomy between June and October 2021. Patient outcomes were compared between delayed and non-delayed surgeries as per the NCEPOD (National Confidential Enquiry into Patient Outcomes and Death) classification. The primary outcome compared was the 30-day post-operative mortality and morbidity determined by the Clavein-Dindo class ≥2.

Secondary outcomes included the time from booking to anaesthesia start time, i.e., time to theatre (TTT), delay in surgery, out-of-hours (OOH) surgery, and unplanned return to theatres.

Results

Of the 103 laparotomies included, 33% were performed over the weekend. The most common indication for emergency laparotomy was bowel obstruction (53.4 %), followed by perforation (28.2%). There was no significant difference in mortality, the TTT (p = 0.218), delay in surgery with respect to the NCEPOD category of intervention (p = 0.401), postoperative length of stay (p = 0.555), number of cases operated OOH as well as unplanned return to theatres. There was a significant difference in the morbidity of patients between the two groups (Clavein-Dindo class ≥2, p = 0.021).

Conclusion

With consistent consultant involvement, an equivalent standard of weekend emergency surgical service can be delivered.

## Introduction

Emergency laparotomy (EL) represents complex and time-critical surgical procedures that are commonly associated with higher mortality and morbidity. The annual National Emergency Laparotomy Audit (NELA) data reflect the changes in clinical practice along with ongoing investigations of care and outcomes [1]. Organisations in the United Kingdom are using NELA data to improve emergency surgical peri-operative care through multi-disciplinary team involvement and developing new patient pathways. NELA modifies annual data collection to reflect changes in clinical practice whilst continuing to investigate processes of care and outcomes, highlighting variation in these [1]. Delays to the theatre inevitably increase the risk of sepsis, deterioration, failure to rescue, and death [2]. Despite this, the timeline between symptom onset and emergency laparotomy is ill-defined with variations in the assessment and management.

A functional, dedicated emergency theatre is essential for acute surgical services [3]. This ensures improving the ease of access to the theatre for acute surgery and a decrease in out-of-hours (OOH) emergency surgery being conducted when there are limited numbers of staff available [4].

Data sets of large national hospitals have shown persistently increased mortality associated with admission on a weekend (WE; Saturday or Sunday) as compared with a weekday (WD) [5,6]. Similar results have been found internationally [5,7,8]. Possible causes are that there is a different case-mix in weekend admissions accounting for excess mortality, or due to reduced service provision at weekends.

A meta-analysis reporting outcomes of over seven million procedures identified that the risk of mortality had a stepwise increase with each day of the week, and the greatest increase in the risk of mortality was observed in weekend emergency admissions (OR 1.27; 95% CI 1.08-1.49) [9]. Factors that affect the "weekend effect" still need to be identified by investigating the possible causes of discrepancies in service delivery, which could help to determine the areas for improvement. To what extent the excess mortality over the weekends is preventable by a change in service still needs to be determined.

In the 2018 NELA report, inconsistent consultant presence out-of-hours and during the weekend was highlighted [10]. Although most patients had been seen in-person or were discussed with a consultant surgeon, only 90% and 66% of patients had intraoperative consultant surgeon and anesthetist presence in and out-of-hours, respectively. More than 30% of >23,000 cases were performed out-of-hours. Importantly, patients who underwent surgery out-of-hours had a higher proportion of patients with a predicted risk of death >10% (55%), compared to in-hours operations (35%). The involvement of experienced decision-making also has an impact on post-operative critical care admissions, which evidently impacts subsequent clinical outcomes.

Data in NELA is heterogeneous, which does not take into account the variations in services across the country. Hence, assessing single centre outcomes may give a better understanding of outcomes against the services provided, thereby assisting in comparing models of delivery and their outcomes between centres. This, in turn, may guide planning services.

We wished to investigate if the weekend affected delays in surgery and the outcomes of the patients undergoing emergency laparotomy at our institute.

Our hospital is a large acute general hospital catering to a population of over 750,000. We perform over 200 emergency laparotomies annually. The surgical department has a consultant-delivered service that covers out-of-hours and over the weekend. With consistent consultant availability over weekends, we would expect fewer pre-operative delays in patients undergoing laparotomies, which would consequently reflect on the outcomes of these patients.

In order to assess these, we conducted a retrospective study of emergency laparotomies performed over a period of five months, from June 2021 to October 2021. The aim of this study was to compare the delays and outcomes of emergency laparotomies performed on weekdays and weekends at a high-volume, large district general hospital.

## Materials and methods

This was a retrospective cohort study of all adults aged ≥16 years undergoing non-trauma, non-vascular emergency laparotomy between June 1, 2021 and October 31, 2021. Data on general surgical take were obtained and compared from a prospectively maintained theatre database on WD and weekends WE.

Our hospital is a large acute NHS district general hospital in England, serving a population of around 750,000 from a wide range of social and ethnic groups across North East London. Polytrauma cases requiring laparotomy were referred to the regional trauma centre; hence, they were not included in our study. Approximately 200 laparotomies are performed annually. The emergency services on weekdays from 0800 hours to 1800 hours are covered by a CEPOD (National Confidential Enquiry into Patient Outcomes and Death) consultant surgeon who covers CEPOD theatres as well as all acute general surgical admissions. From 1800 hours to 0800 hours the next day is covered by another consultant surgeon who is responsible for all acute surgical admissions over the next 24 hours. Weekends are covered by two consultants, one for all admissions on Friday, who covers CEPOD theatre as well as reviews all new admissions on Saturday. The other consultant covers all admissions on Saturday and Sunday while also covering CEPOD on Sunday. All new admissions are reviewed by the consultant surgeon within 12-14 hours of admission.

This arrangement of services ensures enough rest for consultant surgeons, thereby ensuring full engagement in the emergency caseload. Early involvement in the review of unstable or complex patients helps in timely decision-making.

There are four CEPOD theaters; one is a fully staffed, dedicated general theatre running 24 hours a day, all seven days of the week. Another fully staffed theatre is available from 0800 hours to 1800 hours daily, which is shared with urology, gynaecology, and other surgical specialties, while there is a 24-hour trauma CEPOD theatre for orthopedics and a dedicated neurosurgery theatre. All general surgical emergency operations are performed by or under the direct supervision of a specialist consultant general surgeon.

The primary outcome compared was the 30-day post-operative mortality and morbidity determined by the Clavein Dindo class ≥2. Secondary outcomes included the time from booking to anaesthesia start time, i.e., time to the theatre (TTT), delay in surgery, OOH surgery, and unplanned return to theatres.

Data were collected using electronic patient records and all data were anonymized. This included patient demographics, including age and gender. Additionally, the ASA and the Charlson Co-morbidity Index (CCI) [11] were adopted to assess surgical risk. The time to the theatre, date and time of theatre booking (to assess if it was a weekday or weekend), the slot of booking, the number of operations done OOH, pre-operative radiological investigations, post-operative critical care admission, post-operative complications, length of hospital stay, and unplanned return to the theatre were recorded. In addition, the surgeon and anaesthetist involved in the laparotomy were recorded according to whether they were consultants or not.

TTT was defined as the difference between the time of booking and the start of anaesthesia. The booking time was recorded according to the time of the day: daytime (08:01-18:00 hours), evening (18:01-00:00 hours), or night-time (00:01-08:00 hours). Evening and night-time were considered OOH. Timeliness of arrival at the theatre was determined by the NCEPOD classification of intervention as per the booking form.

All data were collected using Microsoft Excel. Continuous data were expressed as the median. Data were analysed using the two-tailed t-test for continuous data and χ2 or Fisher's exact tests for categorical variables. A level of statistical significance was set at a P-value of <0.05. Predictors of mortality and morbidity were analysed using binomial logistic regression analysis.

This study was approved by the divisional governance committee and complied with all local and institutional guidelines on information and research governance. The need for informed consent was waived due to the retrospective study design. The hospital research ethics committee exempted this study from ethics approval.

## Results

During the study period, 110 laparotomies were performed. After excluding vascular and gynaecological laparotomies, 103 patients were included for analysis. Amongst 103 laparotomies, 69 (66.99%) were performed during the weekdays and 34 (33%) over the weekend. Overall mortality was 10.67% and Clavein Dindo class ≥2 morbidity was 42.71%. There was no significant difference between the TTT, delay in surgery with respect to the NCEPOD category of intervention, number of cases operated OOH as well as unplanned return to theatres (Table 1). With respect to the procedure performed, the most common indications for emergency laparotomy were bowel obstruction (53.4%), followed by perforation (28.2%).

**Table 1:** Comparison of patient characteristics and pre-operative investigations *Two-tailed t-test; #chi-sqaure test. Pre-op CT: pre-operative CT scan, TTT: time to theatre, OOH: out-of-ours, CCI: Charlson Co-morbidity Index.

	Weekday	Weekend	p-value
N	69	34	
Age	67(24-94) SD 15	66 (21-90) SD 18	0.974^*^
Gender			
Male	25	21	0.025^#^
Female	44	13	
CCI	3.55	3.705	
≤5	62	28	0.83^#^
>5	6	4	
ASA	2.88	2.94	0.752^*^
<3	3	1	
≥3	66	33	
PRE op CT			
In house radiologist	24	8	0.35^#^
Outsourced	45	26	
Indication			
Perforation	22	7	0.487^#^
Obstruction	35	20	
Others	12	7	
NCEPOD booking			
1	10	5	0.401^#^
2	53	23	
3	6	6	
Time of booking			
Daytime	48	24	0.339^#^
Evening	10	2	
Night-time	11	8	
Delay in TTT	15	12	0.218^#^
No delay in TTT	54	22	
OOH			
Yes	17	8	0.904^#^
No	52	26	
TTT (in minutes)	370.36	594.53	0.387^*^
Consultant anaesthetist present			
Yes	64	29	0.396^#^
No	5	5	

Most patients operated on the weekdays were females (63.8% of females during the weekdays, 61.8% of males on the weekend, p=0.025). However, there were no significant differences with respect to age. The ASA grade and CCI were similar between the two groups. Most CT scans were reported by outsourced consultants in both groups; however, this difference was not significant (Table 1). There was no difference in the indication of surgery, NCEPOD category of intervention, or the time of booking (Table 1).

As depicted in Table 2, there was a significant difference in the morbidity of patients between the two groups (Clavein Dindo class ≥2, 55.9% weekend and 36.2% in weekday operations, p = 0.021). Overall mortality was comparable between the two groups, as was post-operative critical care admission. Amongst the surgeries performed over the weekend, 17.6% were booked during the weekdays (p<0.0001). Amongst the patients who operated on the weekend, there were no unplanned returns to theatres. Consultant surgeons were present for all the laparotomies. The median post-operative length of stay for those operated during the weekday was 9 days and 10.5 days over the weekend (Table 2).

**Table 2:** Post-operative outcomes of patients *Two-tailed t-test; #chi-sqaure test. LOS: length of stay.

Outcomes	Weekday	Weekend	p-value
ITU stay			
Yes	43	19	0.679^#^
No	26	15	
LOS in ITU	6.07	7.21	0.616*
Clavein Dindo			
<2	38 (55.07)	10 (29.41)	0.021^#^
≥2	25 (36.23)	19 (55.88)	
Length of stay (median, in days)	9 (S.D 2.09)	10.5 (S.D 4.19)	0.555^*^
30-day mortality	6	5	0.555^#^

On binominal logistic regression analysis for weekend operations (Table 3), the odds ratio (OR) of overall mortality was 2.72 (CI 0.55-13.38, p=0.22), and morbidity was 3.57 (95% CI 1.32-9.64, p = 0.012) for laparotomies performed over weekends. Higher ASA was associated with a higher risk of mortality (OR 6.86, 95% CI 2.03-2.21, p=0.001) and morbidity (OR 2.75, CI 1.51-5.03, p=0.001) (Table 3).

**Table 3:** Binominal regression analysis of post-operative mortality and morbidity

	Mortality			Morbidity		
	OR	(95% CI)	p-value	OR	(95% CI)	p-value
Weekend	2.7167	(0.551, 13.383)	0.2193	3.5720	(1.323, 9.64)	0.0120
Age	1.0075	(0.952, 1.066)	0.7941	1.0019	(0.967, 1.038)	0.9176
Gender	1.0219	(0.208, 5.026)	0.9787	1.1508	(0.450, 2.941)	0.7691
Slot of booking	0.7516	(0.256, 2.201)	0.6023	0.8492	(0.469, 1.537)	0.5893
OOH surgery	2.8582	(0.564, 14.487)	0.2047	1.5264	(0.527, 4.416)	0.4352
Delay in TTT	0.8799	(0.165, 4.699)	0.8810	0.7267	(0.267, 1.977)	0.5319
Charlson Co-morbidity Index	0.9851	(0.681, 1.425)	0.9364	0.8886	(0.695, 1.136)	0.3465
ASA	6.8606	(2.036, 23.113)	0.0019	2.7516	(1.505, 5.028)	0.0010

## Discussion

Our study aimed to compare the delays and outcomes of emergency laparotomies performed during the weekend as compared to weekdays. We found no significant difference in mortality. However, morbidity was higher in patients operated on over the weekend (Table 2). The numbers of cases delayed were not significantly different between those operated during the weekdays as compared to those operated on the weekends.

The NECPOD report highlighted the delay in diagnosis in patients with acute bowel obstruction due to delays in consultant review. 40.6% of patients experienced a delay in diagnosis as compared to only 15.6% who had timely consultant review [12]. The report recommended that discussion with a consultant should occur within an hour for high-risk patients and within at least 14 hours for others.

Delays in access to operating theatres for emergency surgery have been shown to be independently associated with an increased risk of in-patient mortality and longer length of stay, which consequently result in higher hospital costs. The availability of clinicians and staff was identified as the main reason for delays in emergency surgery [13]. Highlighting the importance of consultant presence for high-risk operations resulted in various organizations implementing changes, which were reflected in the seventh NELA patient report [1]. Both consultant anaesthetists and surgeons' presence during surgery is at 90.1% and has increased from 77.4% (Year 6) to 85.2% out-of-hours (00:00 to 08:00 hours). This represents an improvement in the service provisions out-of-hours. However, there remain wide variations across the various institutions in the NHS.

Consultant input and intra-operative presence were present in 100% of the cases in our study. Pre-operative involvement of senior anaesthetists and critical care teams aided in planning post-operative critical care admissions. In their study, Schneider et al. [14] showed that age, indication of laparotomy, and consultant surgeon presence were independent predictors of delays in EL. Our study demonstrated that consultant presence and timely consultant review prevented additional delays to theatres on weekends, as evidenced by no significant difference in delays between weekdays and weekends. Individual clinical situations determine the decisions regarding the appropriate timing of surgery. Involvement of consultants early in this decision-making guides better planning and timely organization of theatre and staff.

In our study, there was a greater proportion of high-risk patients, as defined by an ASA score of 3 or higher (67.9% compared to 54% in the NELA report of 2021 [1] and 55% in the NELA report of 2019 [15]). This could be attributed to the large population that our hospital provides acute services to. However, despite the high number of high-risk patients, the mortality rate was comparable to that reported in NELA (10.7% v/s 8.72% [1]). The day of surgery did not affect the mortality of the patients.

The Association of Surgeons of Great Britain and Ireland (ASGBI) report in 2007 identified the availability of abdominal CT scans as an area requiring improvement [16]. In our study, all patients had a pre-operative CT scan. All CT scans were reported by a consultant radiologist, with 68.9% being reported by an outsourced consultant. This is much higher than that reported in NELA (68.9% vs 19.1% [1]). This could be attributed to the large number of patients undergoing CT scans OOH.

Multiple studies assessing perioperative factors in emergency laparotomies have primarily focused on mortality as an outcome [17-19]. It is well established that post-operative complications are common after emergency surgeries, which can range from minor to severe complications [20,21]. To address this, we assessed post-operative morbidity as a primary outcome measure.

Higher ASA grade was predictably associated with higher morbidity; however, age did not correlate with it (Table 3). This could be attributed to the relatively small cohort, which could have introduced an element of bias. However, morbidity (as described by the Clavein-Dindo classification) was higher in patients operated on over the weekend. Though not significant, patients operated on weekends had a higher ASA at the time of surgery, which could be a cause of higher morbidity. In-hospital mortality in the WE group was higher than in the WD group, although this did not reach statistical significance.

The delays in the pre-hospital phase of emergency general surgery are ill-defined. This aspect is important and should be assessed and addressed. Doing so can guide planning pathways to improve engagement with primary and pre-hospital care. Access to social networks and healthcare provision probably has an impact on the timeliness of presentation initially as well as on discharge [22].

Our study showed no difference in the number of cases operated OOH over the weekends as compared to weekdays. The consultant supervision of OOH emergency laparotomy was better than that reported nationally (100% vs 85.2%) [1]. In OOH, the surgery decision would be taken by a call consultant and be guided by the clinical condition of the patient. Among patients operated on weekdays, 4.3% had an unplanned return to the operating room, whereas none of the patients operated on WE had an unplanned re-operation.

Although there was a proportionally smaller number of patients operated on out of hours, a small but consistent number of cases required an operation after hours. Some studies have reported higher complication rates in whom emergency intervention is deferred [21,23]. Hence, OOH surgery remains an important part of emergency surgical services.

Trauma cases have protocol-based pathways that have been comprehensively assessed and implemented worldwide. This is following the acknowledgment that the outcomes of critically unwell patients are dependent on time-critical interventions. Acute abdominal pathology has a reported mortality rate of 74% [24], thereby justifying that fast track pathways should be similarly formulated and implemented for emergency general surgery.

Our study showed no significant differences in post-operative LOS and mortality with respect to the day of surgery (WE vs WD). Moreover, recent improvements in healthcare within the UK, including more protocol-based management of sepsis, better access to intensive care beds, and care for patients requiring emergency abdominal surgery, might have controlled the weekend effect [25]. Differences in staffing levels and availability of resources at weekends may explain the observed variability of weekend effects across the world; however, the current literature does not provide adequate evidence to compare the structure of the on-call emergency general surgery team on weekends in different countries and continents. This highlights a need for further studies focusing on differences in staffing levels and available resources during the weekend in emergency general surgery settings at an international level.

The data from our study and the lack of a difference in outcomes between weekdays and weekends adds weight to the proposition that a weekend effect can be modified for emergency laparotomies. A key factor in our study is the consistent weekend consultant presence and the surgical team. Future studies examining multi-centre data on emergency surgical admissions in the UK may confirm or refute this.

The strength of this study was that electronic theatre records were used, thereby providing accurate information. Additionally, our cohort of patients was diverse, from various socioeconomic backgrounds, reflective of varying levels of complexity of cases. Though we had a relatively small patient cohort, it reflects the large volume of emergency laparotomies performed at a single hospital over a relatively short study period. This is relevant as it reflects the well-equipped emergency surgical services to deal with a mixed patient cohort. Our study was limited by the fact that it was a single-centre retrospective cohort, although these data were obtained from a prospectively maintained database. We did not include the data of patients who were deemed unfit for surgical intervention. Based on the results of our study, we report that weekend surgical services provided are equivalent to that of weekdays.

## Conclusions

Laparotomies performed over the weekend were not associated with an increase in post-operative mortality, however, there was an increase in morbidity. Weekend surgery was not associated with an increase in delays to theatres and had a comparable number of OOH surgeries as compared to weekdays. The findings of this study suggest that a good standard of weekend emergency surgical service can be delivered with a consistent consultant presence.
